# Chronology of Global Success: 20 Years of Prof Vallet-Regí Solving Questions

**DOI:** 10.3390/pharmaceutics13122179

**Published:** 2021-12-17

**Authors:** Miguel Manzano

**Affiliations:** 1 Chemistry in Pharmaceutical Sciences, School of Pharmacy, Universidad Complutense de Madrid, Instituto de Investigación Sanitaria Hospital 12 de Octubre i + 12, Plaza de Ramón y Cajal s/n, E-28040 Madrid, Spain; mmanzano@ucm.es; 2 Networking Research Centre on Bioengineering, Biomaterials and Nanomedicine (CIBER-BBN), E-28034 Madrid, Spain

**Keywords:** silica-based mesoporous materials, mesoporous silica nanoparticles, controlled release, stimuli-responsive drug delivery

## Abstract

Twenty years ago, a group of bold scientists led by Prof Vallet-Regí suggested for the first time the use of mesoporous materials as potential drug delivery systems. Without knowing it; these pioneers unleashed the beast of creativity around the world because that original idea has been the inspiration of hundreds of scientific groups for the design of many versatile delivery systems based on mesoporous materials. Because the dream is not the destination, it is the journey, the present review aims to summarise the chain of events that catapulted a small and young research team from the grassroots of academia to the elite of the Biomedical Engineering field.

## 1. Introduction

All great scientific breakthroughs have always been preceded by a question. The more ambitious the question, the more likely the answer was to become a great discovery. So, this whole journey started with a question, with *The Question*: is it possible to introduce drugs into the porous network of mesoporous materials? A question full of audacity and creativity that Professor Vallet-Regí was able to answer through various experiments, and which led her to pioneer the use of mesoporous silica materials (MSMs) for the controlled release of drugs. In fact, the breaking news of what was a new concept at that time, loading mesoporous materials with pharmaceutical agents, brought together the two scientific areas where Prof Vallet-Regí had spent most of her time: Materials Science and Pharmaceutical Technology.

This review analyses the chronological evolution of this discipline through the very same questions that Vallet-Regí’s team has been solving for the last 20 years; such as, (1) What if drug molecules are introduced into the pores of mesoporous materials? (2) Is it possible to actually control the cargo loading and release? (3) Are the drug molecules truly inside the pores? (4) How that technology could be translated to nanoparticles? (5) Can we have real control on drug release through stimuli-responsive systems? (6) Can those nanoparticles be somehow directed towards certain targeted tissues? (7) How all that technology could be assembled in a nanocarrier? Each of these questions arose as the previous one was being answered and boosted the development of a new application of mesoporous materials.

The present review will recall the exciting journey that led the group of Prof Vallet-Regí to travel unknown paths, which promoted her to lead an area of modern medicine where nanotechnology is here to stay: Nanomedicine.

## 2. What about Introducing Drugs into Mesoporous Materials? *From Zero to Hero*

Sometimes fate plays in your favour, and this is exactly what happened to Prof Vallet-Regí when she left her position at the School of Chemistry to pursue a Professorship in the School of Pharmacy at Universidad Complutense de Madrid. As the poet said, “*Fate is the one who shuffles the cards, but we are the ones who play the game*”, and Prof Vallet-Regí was smart enough to bring with her all the Chemistry and Materials Science knowledge to a totally new world for her, focusing her energy on pharmaceutical aspects. Among them, the biopharmaceutical industry has focused on drug delivery systems that can provide a local and sustained release of therapeutic agents over time, while simultaneously protecting them from physiological degradation. By that time, Vallet-Regí’s expertise and research experience was based on zeolites and their structural aspects [1]. Therefore, she was familiar with porous materials and certain applications, such as catalysis and gas separation. However, the move to the School of Pharmacy inspired her to ask herself a question: taking into account that conventional drug delivery systems were based on carriers able to transport drugs when and where they might be needed, *would it be possible to introduce drug molecules into those porous materials to use them as delivery systems?*

The size of the pores of microporous materials (conventional zeolites present extremely narrow pore size distributions in the range of 0.5 nm) seemed to be insufficient for drug loading. Consequently, she pivoted to the use of mesoporous materials that present a pore size range of 2–50 nm, which are large enough to accommodate drug molecules.

Among all available mesoporous materials, mesoporous silica materials are made of inorganic silica, which is a very interesting material because of its good biocompatibility and low cytotoxicity. Concretely, MSMs consist of an amorphous framework of silica with a network of cavities and have been always considered very popular in the area of catalysts. They were reported for the first time by Kuroda et al. in Japan and the Mobil Oil Corporation researchers in the USA back in the 1990s, who named the materials as MCM-n from Mobil Corporation Materials-n [2,3,4,5]. The reason for such recognition relays on the number of key advantages that they present: narrow pore size distributions, with high surface area (*ca.* 1000 m^2^ g^−1^) and great pore volume (*ca.* 1 cm^3^ g^−1^), high density of silanol groups that allow their easy modification with organic moieties, good biocompatibility and reduced toxicity [6]. Those properties are a direct consequence of their synthetic method, which is based on using self-assembled surfactants that act as templates for the silica condensation. Then, removal of surfactant leads to a network of cavities that determine most of the physico-chemical properties of the produced materials that were initially described with electron microscopy by Prof Inagaki and co-workers [7,8].

**Figure 1:**
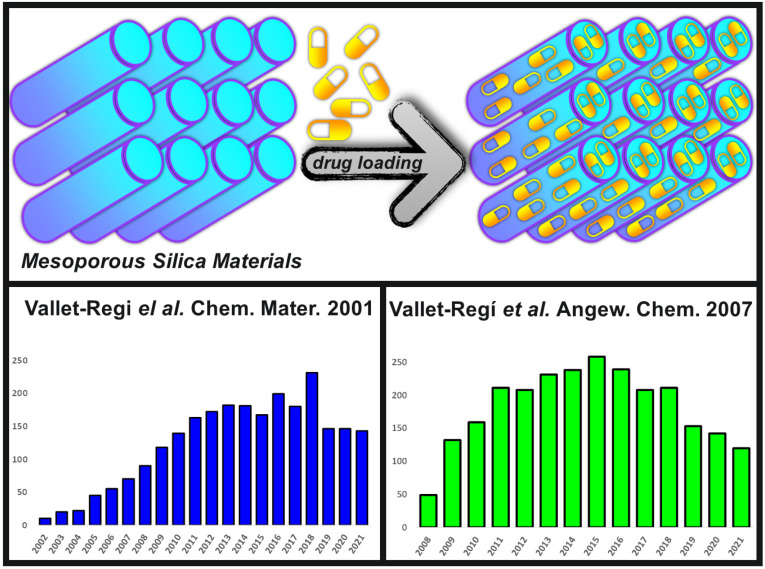
Top: Schematic representation of the original idea of loading drugs into the network of pores of mesoporous silica materials. Bottom left corner: Cites distribution along the last 20 years of the original communication where MSMs were presented as drug delivery systems, adapted from [9], published by American Chemical Society, 2001. Bottom right corner: Cites distribution of one of the most important reviews on this topic reported on Angewandte Chemie after only 6 years of the original research paper, adapted from [10], published by Wiley-VCH, 2007.

Therefore, Prof Vallet-Regí and her newly formed team took into consideration all those features back in the early 2000s and carried the appropriated experiments to demonstrate that those MSMs could efficiently adsorb drug molecules and then release them with predictable kinetics [9]. That new drug delivery system was based on loading ibuprofen into the pores of MCM-41 materials (Figure 1), which are ordered mesoporous silicas with a 2-D hexagonal arrangement of the pores.

This pioneering work on a potential implantable delivery system opened the path to the use of multiple mesoporous structures with improved drug adsorption features, such as SBA-15 (from Santa Barbara Amorphous materials), FDU-5 (from Fudan University ordered mesoporous materials family), or MCF (from Mesocellular Foams materials), as drug delivery systems [11]. Their great surface area, high pore volume and large network of cavities available allow the adsorption of many different biomolecules and pharmaceutical agents into their ordered system of pores. In this sense, there has been and extraordinary growth in the research of these materials owing to the appealing possibilities that they offer to biomedicine [12]. Figure 1 also shows the great number of cites that the original paper on MSMs as drug delivery has been receiving along the years [9], together with one of the seminal reviews from Vallet-Regí and co-workers on this topic [10]. This huge level of citations speaks for itself about the importance of this application, demonstrating that the talent placed in the right place at the right time always pays off.

## 3. Is It Possible to Control the Adsorption and Release Processes? *Power Is Nothing without Control*

After any great and powerful technology might had been developed, there is always the same uncertainty: *power is nothing without control*; a message that perfectly embodies what happened after the pioneering work of proposing mesoporous materials as drug delivery systems. Consequently, there was a straightforward question: *Is it possible to control the drug molecules adsorption and release processes?* The different attempts to answer that question fuelled the research field in this topic at the early 2000s.

In general, the reasons for the great impact of those mesoporous materials in the field of biotechnological research can be found in their textural properties, such as (1) a network full of hollow cavities with narrow pore size distributions (2–20 nm); (2) their high pore volume, favouring the confinement of a large number of drugs or biomolecules; (3) their large surface area, providing a great potential for molecules adsorption; (4) the ordered mesostructure, which leads to a well-ordered pore distribution and the subsequent reproducibility on the drug adsorption and release processes; (5) the presence of Si-OH groups at their surface, both internal and external, bringing the possibility of easy functionalization procedures that could modify the surface of the matrix and, consequently, the interaction with the adsorbed drug molecules; (6) a very robust SiO_2_ framework that permits using a variety of harsh reaction conditions for the different functionalisation processes; and (7) a demonstrated great biocompatibility [13,14].

The high impact of this type of materials in the field of biotechnological research has been powered by the search of answers to the several questions that have been subsequently asked along the way. Among them, an obvious question was: *What kind of molecules could be loaded into the materials?* Alternatively, in other words, *Could all molecules be adsorbed into the pores?* The first thing that needs to be considered to answer those questions is the size of the drug to be confined and the subsequent selection of the adequate matrix depending on its textural properties. In this sense, it was early discovered that the pore diameter would act as a limiting factor of the size of the drug molecule that could be hosted [15]. Thus, in the early stages of this area, it was found that if the drug molecule was smaller than the diameter of the mesopore, it could be confined in the inner part. However, if the size of the molecule was larger than the entrance of the pore, the drug adsorption would only take place at the external surface of the mesoporous material. Therefore, it was early found that the pore size determines what type of molecule can be loaded, acting as a size-selective adsorption parameter [11]. This effect was observed when loading large biomolecules, such as certain proteins, that were on the boundaries of the mesopores sizes [16,17].

Once the size was not a problem anymore because the drug molecules were small enough to fit into the pores, the next important question arose regarding the stability of those molecules inside the pores. When the drug was too small in comparison with the diameter of the pore, most of the molecules could not be retained inside and only a small portion of them could interact with the mesopores inner surface walls. Thus, the most important factor that rules the amount of molecules that could be retained inside the pores is the specific surface area of the host matrices. This effect was observed by the Vallet-Regí team when loading alendronate, a potent bisphosphonate with poor intestinal adsorption conventionally employed in osteoporosis treatments, into MSNs with different surface areas [18]. Both matrices, MCM-41 and SBA-15, present the same mesostructure but different surface areas, *ca.* 1200 and 700 m^2^ g^−1^, respectively. MCM-41 loaded almost twice the amount of drug in comparison with SBA-15, demonstrating that the larger the surface area, the greater the loading capacity.

The pore volume was also found to be another key parameter to take into consideration when quantifying the total loading or when loading large-volume molecules, such as the case of certain proteins.

However, the functionalisation of the pore walls is what really influences the adsorption and retention capacities of these MSMs [19]. The reason for that was the fact that drug adsorption into mesoporous channels is a surface phenomenon that might be governed by the chemical interaction between silanol groups from the silica and the functional groups of the adsorbed guest molecules. Therefore, those interactions could easily be turned through the appropriated functionalisation of the silica walls of the host matrices, improving their retention capacity. Thus, the organic functionalisation of the pore walls should be selected depending on the drug molecule that will be adsorbed, taking into consideration the different functional groups of that guest molecule [20]. Vallet-Regí team has been exploiting this functionalisation process for the last few years to increase and retain many different guest molecules, such as amino acids [21], drugs [22,23], or other type of biomolecules [24].

As it has been commented above, once that the question about the possibility of controlling the loading process was answered, the next question was immediately asked: *Is it possible to control the cargo release?* Researchers of this area, including Vallet-Regí team, employed the same tools previously employed for controlling the molecular adsorption to try to control the release kinetics of the payload. Thus, certain textural properties of the host matrices were found to play an important role in the release kinetics of the adsorbed cargo molecules [25]. Among them, pore diameter was found to have a strong influence on the release rate as observed in an experiment where different MCM-41 matrices were produced with different pore sizes and evaluated as ibuprofen delivery systems [26]. The reduction of the pore diameter led to a decrease in the drug release rate, as initially expected.

The surface area was also a strong effect on the release kinetics because the molecular retention of the cargo molecules depends on the available surface to interact with it. Therefore, the higher the surface area, the slower the release because of the stronger interaction with the pore walls surface. This effect was observed by Vallet-Regí and co-workers when analysing the different release kinetics of alendronate (first and zero order kinetics) from different matrices (MCM-41 and SBA-15, respectively) with different surface areas (*ca.* 1200 and 700 cm^2^ g^−1^, respectively) [18].

However, the functionalisation of the silica walls was found along the years to be the most important parameter to control the cargo release [27]. In this regard, a careful selection of the organic moiety could increase the attractive host-gest interactions and, consequently, slow down the release kinetics [28]. This effect was observed with many different types of functionalised matrices adsorbing a variety of different pharmaceutical agents [29,30,31,32,33].

## 4. Are the Drug Molecules Actually Loaded inside the Pores? *The Truth Machine*

One of the main benefits of using mesoporous silica materials as drug delivery systems is derived from the efficient protection that silica will provide to the encapsulated species. In fact, the drug molecules loaded inside the mesopores should be protected from the harsh environmental factors that can be found in living systems, such as enzymatic degradation or extreme pHs. However, there was a question flying around from the very beginning of this technology, *Are the adsorbed drug molecules actually located inside the mesopore channels?*

Up to 2010, the location of the particles was deduced from the sum of some indirect characterisation techniques, such as Fourier transformed infrared and X-ray fluorescence spectroscopies, N_2_ adsorption, elemental analysis and/or thermogravimetry. However, Prof Vallet-Regí together with her colleague Prof González-Calbet from the *Centro Nacional de Miscroscopía Electrónica* were able to clear the air answering the question of the location of the cargo for the first time ever owing to the use of electronic microscopy [34]. Scanning transmission electron microscopy (STEM) with spherical aberration correctors incorporated allowed for the first time the direct evidence of the molecules confinement into the inner part of the mesopore channels. Once again, Prof Vallet-Regí pioneered the application of a technology (STEM) that permits performing analyses with enough resolution (atomic level) to distinguish between pore walls made of silica and pore space where the adsorbed drug molecules were confined. Owing to the application of STEM to the mesoporous-based drug delivery technology, the question related to the location of the cargo was never asked again, confirming for the first time that adsorbed cargo molecules were actually into the inner part of the mesopores.

## 5. Can We Make This Technology Work in the Nanoscale? *From Macro to Nano*

As one would expect, the excellent textural properties of mesoporous silica matrices together with their potential applications as drug delivery systems rapidly inspired their translation from bulk to the nanoscale dimension [35]. The reason for that relays on the unique physicochemical properties that nanoparticles can offer to drug delivery technologies, including more beneficial pharmacokinetic profile, improved drug solubility, increased drug stability, superior cellular trafficking and greater control over the timing and location of the therapeutic action and release and thereby reducing toxicity.

Therefore, it did not take long to develop mesoporous silica nanoparticles (MSNs) to be used as drug delivery nanosystems by many research groups, where Victor Lin’s contribution should be highlighted [3,36,37,38,39]. Unfortunately, Victor Lin passed away in 2010 at the age of 43, but he will be remembered by his seminal contributions to the development and applications of mesoporous silica nanoparticles, which is a term that he coined to describe silica nanoparticles with a well-defined and controllable morphology. Victor Lin also demonstrated some interesting applications of MSNs, such as those in nanomedicine, being the pioneer in validating the intracellular applications of MSNs as drug delivery systems [40,41].

The literature is full of different methods for the synthesis of MSNs, but, in general, all of them relay in three basic processes: (1) The use of the sol-gel method for producing the silica material; (2) the employment of surfactant molecules as structure directing agents for producing the mesostructured materials; and (3) the highly diluted conditions inspired in the Stöber method to obtain spherical nanoparticles [42,43,44]. The conventional synthetic process normally starts with the surfactant being initially dissolved in water at basic pH. A careful selection of the type of surfactant together with the dissolution conditions, in terms of temperature and concentration, would have an important effect on the self-assembly procedure and, thereby, on the final mesostructure of the nanoparticles. Then, the selected silica precursor might be added dropwise and owing to the sol-gel process, hydrolysis and condensation of that precursor take place around the surfactant molecules previously dissolved. The silica network produced forms a colloidal solution, *sol*, that gradually evolves towards a *gel* or discrete particles, depending on the synthesis conditions [45,46]. During this stage, it is important to keep very dilute conditions, which favours the production of monodispersed spherical silica particles [45]. As the *sol-gel* process evolves, the initially formed droplets of silica would be gradually transformed into solid nanoparticles. Finally, the surfactant molecules that worked as structure template could be removed through either solvent extraction or thermal degradation, leading to pure silica MSNs.

The outstanding properties of MSNs fuelled the development of new advanced and multifunctional materials that could be applied in a wide range of biotechnological applications, such as cancer treatment, infectious treatment and certain bone diseases [47]. Moreover, MSNs have also been explored as imaging nanosystems, because there are many different types of dyes and/or contrast agents that have been incorporated into those nanocarriers for diagnostic applications [48].

A key parameter of nanoparticles in biomedicine is the route of administration, including intravenous, subcutaneous or localised injections in the targeted area. The latter option normally leads to a fast distribution of the nanoparticles throughout the whole organism, although there could be some issues, such as their recognition by the mononuclear phagocyte system that decreases the efficiency of any potential treatment. The functionalisation of the nanoparticles with certain moieties, such as poly(ethylene glycol), PEG, might help to reduce the amount of adsorbed proteins and, therefore, reduce the protein corona effect and increase the circulation half-life [49]. This approach has been validated in murine models, where MSNs rapidly accumulated in the lung, spleen and liver after vein injection, while when those MSNs were decorated with PEG, they became stealth and showed increased circulating half-life [50].

**Figure 2:**
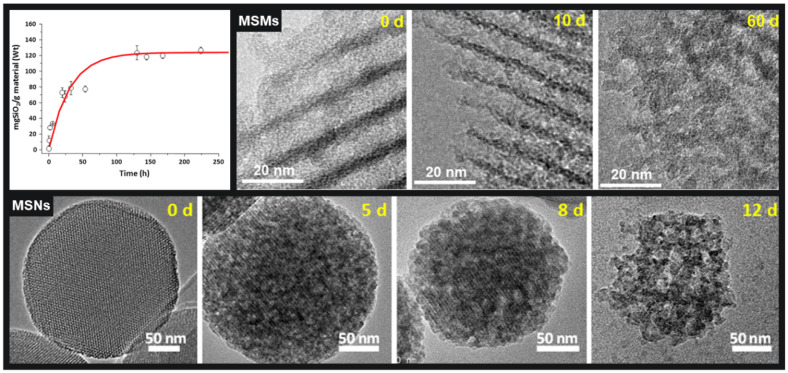
Top left corner: Silica dissolution profile of pure MSMs in physiological medium (Phosphate Buffer Solution, PBS); top right corner: transmission electron microscopy images of the degradation process of MSMs in PBS at different time points; Bottom: transmission electron microscopy images of the degradation of MSNs in PBS at different time points. Adapted with the permission from [51], published by Springer Nature, 2017.

Another important parameter of highly porous nanoparticles such as MSNs is their hydrolytic stability, specially taking into account that all those potential biomedical applications above mentioned would take place under physiological fluids. Therefore, the next question regarding the degradation rate of MSNs in biological environments quickly raised: *Are those MSNs stable enough to be employed in a real scenario?* In this sense, Prof Vallet-Regí has been recurrently wondering about the degradability of these particles under physiological conditions [51]. This is important because the potential degradability of any drug delivery system is a key parameter in their possible successful translation to the clinic. Moreover, it is surprising that despite all the attention that these materials have been having as drug delivery systems, little or no attention was paid until recently to evaluate the degradation of those materials under relevant biological environments. In this sense, the dissolution of bulk sol-gel silica matrices in physiological fluids have been investigated by different authors [52,53,54,55,56,57]. The dissolution of those MSMs was found to follow a two-stage mechanism, with an initial burst surface erosion that was followed by a slow bulk degradation, as it can be observed in Figure 2 for the dissolution of SBA-15 materials [55]. It was found that there are several parameters that might influence the degradation of bulk mesoporous silica matrices in bulk, such as, textural and structural properties, silica condensation degree, possible surface organic modification and, obviously, the physiological medium where the dissolution process might take place.

However, the number of studies exploring the eventual dissolution of MSNs is a bit smaller in comparison with the raised interest in drug delivery systems [58,59,60,61,62]. Similar parameters controlling the dissolution to those observed for bulk mesoporous silica were found for the nanoparticles, such as textural parameters, organic functionalisation and different physiologically relevant media employed for the studies (Figure 2). Interestingly, particle size was not a key effect since dissolution profiles were observed to be pretty much similar, independent of the nanoparticles sizes [63,64]. In conclusion, and although nanoparticle degradation might take place faster than in bulk, Vallet-Regí group found that the rate at which dissolution normally takes place could be controlled by the careful design of some key parameters of the MSNs, such as textural properties and/or surface organic modifications. Consequently, the nanocarriers degradation can be tuned through a careful selection of certain synthetic and post-synthetic parameters, positively answering the initial question of MSNs being stable enough to be employed in real scenarios.

## 6. Can Drug Release Be Controlled through Stimuli-Responsive Mechanisms? *On Demand Services*

Since the very beginning of introducing nanoparticles into the channels of the mesopores, the question has been always there: the accessible porosity of mesoporous materials meant that it was very simple to include drug molecules into their network of cavities, but it was also very easy for those cargo molecules to diffuse out when placed in biologically relevant solutions. So, *would it be possible to control the cargo release?*

**Figure 3:**
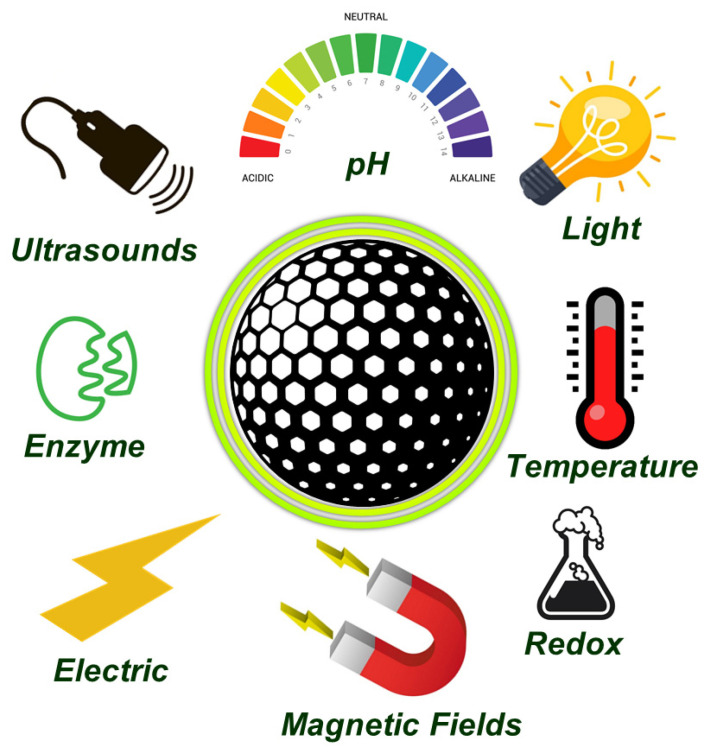
Schematic representation of loaded mesoporous silica nanoparticles surrounded by the different stimuli, both internal and external, that could be used to trigger the cargo release.

The team of Prof Vallet-Regí has spent most of the last 10 years developing on demand nanosystems employing MSNs, which can be approached from different perspectives, such as grafting sensitive gates to the pore entrances or nanoparticle coating with detachable shells [65,66,67,68,69]. Then, the pore entrances could be opened under the operation of certain stimuli, that can be internal or external. The former stimuli are typical from the treated pathology where certain relevant biomarkers might be overexpressed or downregulated, including pH, redox potential and enzymes, among others. On the other hand, external stimuli can be remotely applied, and include magnetic fields, ultrasounds, electrical fields or light (Figure 3).

### 6.1. Magnetic Fields Responsive Mesoporous Nanoparticles

Magnetic fields are popular stimuli in nanomedicine since they can increase locally the internal temperature when employing an alternating magnetic field [70]. In this regard, this was the first stimulus that Prof Vallet-Regí team approached back in 2011 [71]. Mesoporous nanoparticles were functionalised with a single DNA strand and the pores were loaded with the selected cargo molecules. Separately, magnetic iron oxide nanoparticles of *ca.* 5 nm were functionalised with the complementary DNA sequence, so when the later nanoparticles were added to the former MSNs, the DNA hybridisation provoked the blockage of the pore entrances owing to the iron oxide nanoparticles. The magic of it was that the selected DNA presented a melting temperature of 47 °C, so when the nanocarriers were exposed to an alternating magnetic field, the iron oxide nanoparticles increased the local temperature and the subsequent double-stranded DNA melting led to the aperture of the pore entrances and to the cargo release on demand. Many other approaches have been explored incorporating iron oxide nanoparticles into the network of MSNs and covering the pore entrances with thermoresposive polymers, so the application of certain magnetic fields produce an increase of temperature and the subsequent change in the polymer conformation that leads to the cargo release [72].

### 6.2. Light Responsive Mesoporous Nanoparticles

The research from Vallet-Regí team on the use of light with different wavelengths (ultraviolet, visible or near-infrared) has contributed to the increase of popularity of this type of triggers in the last few years [73]. A simple proof of concept was developed back in 2015 when MSNs were coated with a protein shell through a photosensitive linker. Thus, after the cellular internalisation of the MSNs, the application of ultraviolet light triggered the cargo release inside the cytoplasm of the cells. The low penetration capacity of that type of light was improved using a different photo-linker sensitive to visible light [74]. In a more recent approach, her team was able to prepare MSNs sensitive to near infrared, which provoked local hyperthermia and generated toxic reactive oxygen species for antivascular therapeutics in cancer treatment [75].

### 6.3. Ultrasounds Sensitive Mesoporous Nanoparticles

Ultrasounds (US) was the new kid on the block of the Vallet-Regí Lab regarding external stimuli, and it went straight to *top of the pops* owing to their penetration capability without causing damage [62,76,77,78]. The first design was simple and smart, based on decorating the surface of mesoporous nanoparticles with a polymer sensitive to temperature and US to close the pore entrances. Once the nanoparticles might have reached their targeted tissue, the application of US from an equipment conventionally used in rehabilitation clinics, cleaved some bonds and switched the hydrophobicity and conformation of the polymer, opening the pore entrances and triggering the cargo release quite effectively.

### 6.4. pH Sensitive Mesoporous Nanoparticles

Among the available internal stimuli, pH is perhaps one of the most employed triggers because of the different pH values between healthy tissues and tumours. The reason for that is the immense rate of glycolysis in cancer cells that leads to a high production of lactic acid with the subsequent acidification of the tumour tissue. This difference on the pH has inspired many groups to develop pH sensitive gates of the pores to ensure the release only at acidic environments. Prof Vallet-Regí kept track of developing her own pH responsive system based on a gelatin to transport topotecan, a potent cytotoxic drug employed for cancer treatment that degrades at physiological pH [79]. Her team also developed for the first time a nanocarrier based on MSNs decorated with self-immolative polymers, which are a new type of polymers that disassemble from head to tail under certain triggers [80,81,82,83]. A careful design of the trigger sensitive to pH allowed the development of MSNs that release their cargo only under acid pH owing to the disassembly of the polymer that was closing the pore entrances at physiological pH.

### 6.5. Redox Sensitive

Although tumour tissues are known to present higher concentration of glutathione (GSH) than healthy tissues, different levels of GSH have also been observed throughout the organism, such as low levels in blood or extracellular matrices or high levels inside the cells, particularly in the mitochondria and cytosol. Therefore, GSH sensitive bonds, such as disulphide groups, have been developed as linkers to engineer many different gatekeepers to close the pore entrances of previously loaded MSNs. In this way, MSNs could travel along the bloodstream without leaking the cargo because of the low GSH levels in blood. However, once the nanoparticles might be internalised, the high levels of GSH in the cytosol would break the sensitive linkers opening the mesopores entrances and triggering the cargo release only inside the cells [84]. Prof Vallet-Regí research team has also worked with this type of stimulus through the development of MSNs decorated with a redox-responsive gatekeeper based on self-immolative polymers so the pore gates were opened only inside the cells where GSH levels were relatively high [85].

## 7. Can MSNs Be Targeted towards Specific Tissues? *A Fantastic Voyage*

The *magic bullet* is a well-known concept in medicine that was originally based on killing specific microbes without harming the body itself. The application of this approach to nanomedicine gave rise to targeted nanoparticles able to deliver the therapeutic agents only in the required specific tissues, which are of particular importance in potential cancer therapies to avoid side effects and damage to healthy tissues. Prof Vallet-Regí contributed to the development of targeted MSNs to specific tissues with different approaches, both through passive and active targeting. In this sense, passive targeting, that is based on the accumulation of nanoparticles in tumour tissue due to the permeability of tumour vessels, was exploited for the development of MSNs able to overcome different biological barriers [85].

On the other hand, active targeting, that is based on grafting to the surface of nanoparticles certain ligands that might present high affinity towards specific membrane receptors overexpressed in tumour cells, has also been tackled by Prof Vallet-Regí. Among all the targeting ligands grafted to MSNs, folic acid and different peptides have been explored to direct the nanoparticles and their cargo towards cancer cells [86,87,88].

A very imaginative idea for transporting the mesoporous nanocarriers towards tumour tissues was using cells with migratory properties towards tumours as MSNs carriers. In this sense, human Decidua Mesenchymal Stem Cells present tumour-tropic properties so doxorubicin loaded MSNs were internalised into those cells, and tumour cells death was observed, representing a very promising platform for the potential treatment of cancer [89,90].

As it can be observed in other manuscripts of this special issue, Prof Vallet-Regí has also dedicated a lot of energy to the development of different biomaterials for bone tissue regeneration. In this sense, bone has been always the target of her research, so she also contributed to the development of MSNs with bone targeting abilities for the potential treatment of bone diseases, such as bone cancer treatment [91], osteoporosis [92] and bone infection [93]. 

## 8. How to Assemble All That Technology in a Single Nanocarrier? *Lego-like Modular Building Blocks*

Because all roads lead to Rome, it was only a matter of time before Prof Vallet-Regí and her group from the School of Pharmacy would direct their research on nanoparticles towards biomaterials science and tissue engineering. In fact, her Smart Biomaterials Research Group has been extensively working in different approaches related to bone tissue engineering and different bone pathologies. Since there are many excellent reviews on the former, Refs. [94,95,96,97,98,99,100] here we will focus in the later approach, that has been the focus of her research for the last 5 years thanks to the European Commission funding through an Advanced Grant. In this project, some of the most complex bone diseases, such as bone cancer, osteoporosis or bone infection, have been tackled with MSNs [47]. It has been approached designing multifunctional nanodevices based on MSNs acting as nanoplatforms for the assembly of the different building blocks such as, the cargo drug molecules, the nanoparticle itself, the stimuli-responsive mechanism and the targeting moieties [101].

**Figure 4:**
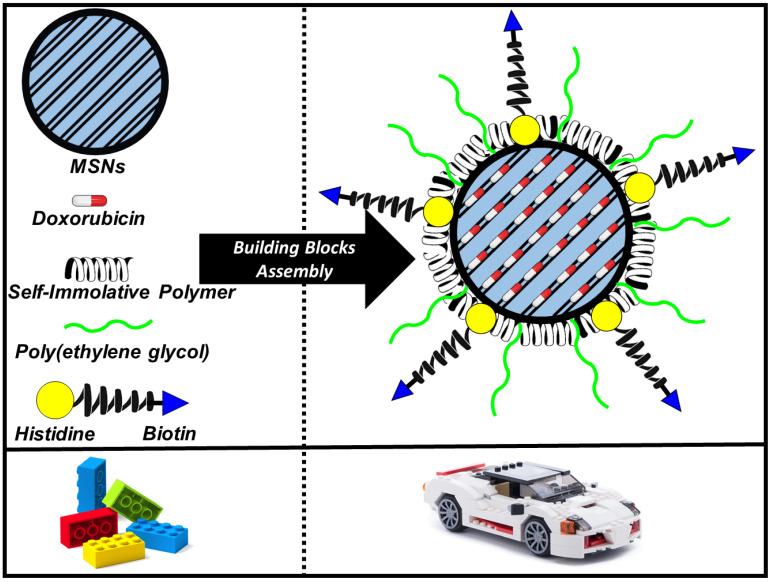
Schematic representation of the building blocks assemble for developing a stimuli-responsive smart nanocarrier. MSNs: nanocarrier platform; Doxorubicin: cytotoxic drug; self-immolative polymer: redox-responsive polymer; poly(ethylene) glycol: stabilising agent in physiological media; Histidine: endosomal escape; Biotin: active targeting agent.

Those building blocks should be carefully assembled for the design of nanocarriers able to overcome the different biological barriers that they might encounter along their journey. Actually, those biological barriers have been found to be a bottleneck for drug delivery nanoparticles to reach the bedside despite the huge amount of research carried out in the laboratory. An admirable example of building blocks assembled into a versatile nanocarrier to overcome different biological barriers has been recently published by Vallet-Regí and co-workers in a collaboration with Prof Tamanoi´s Lab from Kyoto University [85]. This approach was based on a redox-responsive nanocarrier employing MSNs as the main platform decorated with a versatile molecule containing biotin as targeting molecule and histidine as endosomal escape agent, linked to the nanoparticle with a redox-responsive self-immolative linker and loaded with a cytotoxic drug (Figure 4). Thus, the careful selection of different modular building blocks allowed assembling a great amount of technology in a unique versatile nanocarrier able to specifically recognise tumour cells, selectively accumulate into tumours, escape from the endo-lysosomes and kill those cells in different 2D and 3D tumour models.

During the last five years of research in Vallet´s group, all those available building blocks have been adapted and combined in a Lego-like system for building a library of different pieces the treatment of different complex bone diseases.

### 8.1. MSNs for the Potential Treatment of Bone Cancer

Some conventional treatments of cancer, such as chemotherapy, radiotherapy and/or surgery, present several drawbacks, such as lacking tumour tissue selectivity, which leads to non-specific drug distribution and many side effects. In the last few years, nanoparticles have raised as powerful weapons against cancer thanks to their capacity of drug encapsulation and, therefore, side effects reduction [102]. Among all the available nanoparticles explored for cancer treatment, MSNs offer several advantages, such as the robustness of the silica framework that allow almost any chemical modification on their surface, and their outstanding textural properties, that favour their high cargo loading capacity. Thus, it is possible to design a nanoplatform based on MSNs for the specific treatment of bone cancer, and thanks to the careful selection of the different available building blocks, Vallet-Regí and team were able to develop a nanosystem based on the sequential targeting of bone tumours or bone metastases that can also be implemented into any other form of nanomedicine for the treatment of different diseases [88]. This building block system is based on sequential targeting agents: alendronate, which is a bisphosphonate that presents high affinity towards bone tissue, followed by a peptide that contains a cathepsine-K cleavable sequence followed by a RGD motif that is known to favour the selective internalisation into osteosarcoma cells. Thus, alendronate ligand would contribute to the accumulation of the MSNs into the bone tumour tissue, and then the well-known overexpression of cathepsine-K in those tumour environments would cleave the peptide sequence exposing the RGD motif, which would trigger the preferential uptake of the MSNs by the tumour cells. In a similar approach, RGD was exploited as a motif to recognise endothelial cells to target MSNs to the tumour endothelium of fibrosarcoma in a multimodal building block nanoplatform [75]. Another example of a modular building block developed by Vallet-Regí and co-workers was based on shielding the positive charged surface of previously amine decorated MSNs with a cleavable PEG [76]. The application of external Ultrasounds would remove the PEG shield and expose the positive charged MSNs that could be internalised by tumour cells.

### 8.2. MSNs for the Potential Treatment of Osteoporosis

Osteoporosis is a disease characterised by a reduced bone mass and bone tissue microarchitecture deterioration that predominantly affects aged women. Its origin can be found in the imbalance of the bone remodelling process, which is meant to remove old bone and create new bone. Current osteoporosis treatments present many limitations, so the use of different strategies based on drug delivery systems have been explored. Among them, the group led by Prof Vallet-Regí developed back in 2006 mesoporous silica matrices for the delivery of alendronate, a bisphosphonate conventionally employed for the treatment of osteoporosis, in an attempt to improve its bioavailability in bone tissue [18,103]. In a similar approach, an osteogenic peptide called osteostatin was loaded into the pores of mesoporous matrices for stimulating osteoblastic growth in vitro and in vivo [104,105,106] Very recently, Vallet-Regí and group have explored the use of MSNs for the potential treatment of osteoporosis using small interfering RNA (siRNA) able to knockdown a specific gene: SOST [107]. This gene encodes a protein, sclerostin, that is overexpressed in osteoporotic situations and responsible for inhibiting the Wnt/β-catenin pathway, which is a major signalling pathway that regulates bone development and remodelling. Therefore, overexpression of the protein leads to a decrease in osteoblast differentiation, so MSNs targeted silencing the gene coding for that protein. This promising approach has demonstrated to work in an ovacteromised female mice model both injected into the bone marrow and subcutaneously, in an attempt to explore a systemic route of administration, where the expression of certain osteogenic markers was observed and the improvement of some structural and micro-architectural properties of the bone was achieved [92].

### 8.3. MSNs for the Potential Treatment of Bone Infections

The increased prevalence of bone infections in developed countries is directly related with the ageing of the current society, and the subsequent increase on the usage of implantable medical devices that could potentially be contaminated by bacteria. In this regard, the inappropriate use of certain antimicrobials is fuelling the number of cases of drug-resistance bacteria, which will become a serious problem in less than 2 decades. The group of Prof Vallet-Regí has also been working in the last few years engineering multifunctional mesoporous silica nanomatrices capable of preventing bacterial adhesion and biofilm formation, which is a protective matrix that endows bacteria with resistance to antibiotics and immune systems. Additionally, those matrices were able to release antimicrobials in the infected bone tissues [108]. Different modular building blocks have been employed using MSNs as core platform for developing positively charged nanocarriers, that increase their affinity towards the negatively charged biofilm and bacteria [109,110]. Similarly, lectins have been also included into this technology as nanoparticle ligand to increase internalisation into the biofilm, which increase the antibacterial effect of the nanocarriers by itself, and which could be emphasised loading and releasing different types of antibiotics [111].

The modular building block strategy pushed to its limits is the incorporation of MSNs into scaffolds that mimic bone tissue and contribute to its regeneration in certain situations. The role of the MSNs in this type of platforms is their drug loading and controlled release capabilities, as it has been described throughout this review [35].

## 9. Conclusions

The present review has described the path of success of a great researcher, Prof Vallet-Regí, who managed the necessary ingredients, including talent and hard work, at the right place and at the right time. Now that the sunset of her career is approaching, it is only right and necessary to recapture the global success story of the last 20 years. This review, told from the inside, has tried to acknowledge her successful career and, more importantly, to inspire young scientists to never stop believing and to always pursue their dreams, because sometimes they do come true.

## Data Availability

Not applicable.
